# Integrated 16S and metabolomics revealed the mechanism of drought resistance and nitrogen uptake in rice at the heading stage under different nitrogen levels

**DOI:** 10.3389/fpls.2023.1120584

**Published:** 2023-04-04

**Authors:** Changhui Sun, Runnan Wang, Guoping Tang, Shuo Cai, Hong Shi, Fangping Liu, Hengwang Xie, Jinyan Zhu, Qiangqiang Xiong

**Affiliations:** ^1^ Jiangsu Key Laboratory of Crop Genetics and Physiology, Agricultural College of Yangzhou University, Yangzhou, China; ^2^ Jiangsu Key Laboratory of Crop Cultivation and Physiology, Agricultural College of Yangzhou University, Yangzhou, China; ^3^ Jiangxi Academy of Agricultural Sciences Rice Research Institute, Nanchang, China; ^4^ Jiangxi Irrigation Experiment Central Station, Nanchang, China; ^5^ Jiangsu Co-Innovation Center for Modern Production Technology of Grain Crops, Yangzhou University, Yangzhou, China

**Keywords:** rice, heading date, nitrogen, drought, 16S rRNA, metabolomics

## Abstract

The normal methods of agricultural production worldwide have been strongly affected by the frequent occurrence of drought. Rice rhizosphere microorganisms have been significantly affected by drought stress. To provide a hypothetical basis for improving the drought resistance and N utilization efficiency of rice, the study adopted a barrel planting method at the heading stage, treating rice with no drought or drought stress and three different nitrogen (N) levels. Untargeted metabolomics and 16S rRNA gene sequencing technology were used to study the changes in microorganisms in roots and the differential metabolites (DMs) in rhizosphere soil. The results showed that under the same N application rate, the dry matter mass, N content and N accumulation in rice plants increased to different degrees under drought stress. The root soluble protein, nitrate reductase and soil urease activities were improved over those of the no-drought treatment. Proteobacteria, Bacteroidota, Nitrospirota and Zixibacteria were the dominant flora related to N absorption. A total of 184 DMs (98 upregulated and 86 downregulated) were identified between low N with no drought (LN) and normal N with no drought (NN); 139 DMs (83 upregulated and 56 downregulated) were identified between high N with no drought (HN) and NN; 166 DMs (103 upregulated and 63 downregulated) were identified between low N with drought stress (LND) and normal N with drought stress (NND); and 124 DMs (71 upregulated and 53 downregulated) were identified between high N with drought stress (HND) and NND. Fatty acyl was the metabolite with the highest proportion. KEGG analysis showed that energy metabolism pathways, such as D-alanine metabolism and the phosphotransferase system (PTS), were enriched. We conclude that N-metabolism enzymes with higher activity and higher bacterial diversity have a significant effect on drought tolerance and nitrogen uptake in rice.

## Introduction

Dramatic changes in global climate will increase the intensity and frequency of drought events. The phenomenon of persistent drought has become more frequent in the southern region of China (Xiong et al., 2020). A record-breaking heat wave hit the Yangtze River valley during the boreal summer of 2022 (Zhang et al., 2023). Meteorological drought in the Yangtze River Basin has continued to develop, and the situation of rice production has been relatively severe. The lack of water not only inhibits the growth and development of crops but also affects the absorption, utilization and distribution of nutrients in crops, resulting in changes in the accumulation of dry matter and nutrients in various organs (Li and Yang, 2017; Asadi and Eshghizadeh, 2021). When encountering drought stress, it is generally believed that drought first acts on the root system, affecting the morphological structure and physiological activity of the root system and changing its ability to absorb water and nutrients (Steinemann et al., 2015; Li et al., 2020). At the same time, there are certain effects on the root microbial community and metabolites. Due to the reduced cost and increasing throughput of next-generation sequencing, major advances in characterizing these communities have recently been achieved, mainly through the use of amplicon sequencing of the 16SrRNA gene. Compositional analysis of these communities is important to develop tools that will let us manipulate root-associated microbiota for increased crop productivity (Edwards et al., 2018). Studies have shown that the overall response of the bacterial microbiota to drought stress is taxonomically consistent across soils and cultivars and is primarily driven by an enrichment of multiple Actinobacteria and Chloroflexi, as well as a depletion of several Acidobacteria and Deltaproteobacteria (Santos-Medellín et al., 2017). The energy metabolism pathway, carbon fixation in the photosynthetic organism pathway, carbohydrate metabolic process, and reactive oxygen species metabolic process functions have a certain correlation with drought stress (Xiong et al., 2019a).

Water and fertilizer are the two main factors that limit agricultural production. The mutual restriction of water and fertilizer affects the growth of crop roots (Gu et al., 2016). Therefore, when crops encounter arid environments, the growth and metabolism of crops are also limited by nutrients. Soil moisture affects not only the transformation and migration of nutrients in the soil but also the absorption, transport and distribution of nutrients by the plant by affecting the metabolic process of the plant. Nutrients also affect plant water status and the progression of drought stress (Gonzalez-Dugo et al., 2010; Shao et al., 2019). Among them, nitrogen (N) is one of the main elements of amino acids, chlorophyll, nucleic acids, lipids and many intermediate metabolites. N plays an important role in plant growth and agricultural production (Xiong et al., 2019c), further affecting the enrichment of root microorganisms. Studies have shown that compared with no N fertilizer treatment, N application causes the N-fixing bacterial community in rice roots to aggregate and significantly increases the relative abundance of Methylosinus in rice roots (Liu et al., 2021). Appropriate coupled regulation of water and N can create a good rhizosphere environment for rice growth (Xu et al., 2017a), and mild dry-wet alternating irrigation and moderate N increase the enzyme activity in rice rhizosphere soil and increase the number of microorganisms in the soil (Xu et al., 2017b). N deficiency at the tillering stage and N compensation at the young panicle differentiation stage increase the activities of nitrate reductase and glutamine synthase (Xiong et al., 2018). In this study, Yangda 3 (late-maturing medium *japonica*) was used as the experimental material. The barrel cultivation method was used to apply no drought and drought stress at the heading stage. Three different N application levels were set. The study aimed to explore the effect of different water and N applications on the effects of the SPAD value of rice leaves, dry matter mass of rice organs, N content of rice organs and soil, N accumulation of rice organs, N-metabolic enzyme activities of roots and soil urease activities. Furthermore, integrated microbiology and liquid chromatography-mass spectrometry (LC-MS) metabolomics analysis was used to reveal the N uptake mechanism of rice in response to drought stress under different N levels. This study provides a theoretical reference for improving rice drought resistance and N utilization efficiency.

## Materials and methods

### Experimental material

The late-maturing medium *japonica* conventional *japonica* rice variety Yangda 3 (Rice No.20210062, Nanjing 46 irradiated early-maturing line/Suxiangjing 3) was used as the experimental material. The rice seeds were provided by the College of Agriculture, Yangzhou University.

### Cultivation method

The experiment was carried out in 2021 in the on-campus potting field of the College of Agriculture, Yangzhou University. The barrel planting method was adopted. The height of each barrel was 25 cm, the outer diameter of the upper part of the barrel was 28 cm, and the inner diameter of the bottom of the barrel was 20 cm. The seeds were subjected to a sterilization treatment and sown on May 26, 2021, then the rice plants were transplanted on June 18. Seedlings with good and consistent growth were selected for transplanting, with 3 holes per barrel, 2 seedlings per hole, and 15 kg of air-dried soil per pot. After transplanting, water, disease and pest management were carried out according to high-yield cultivation methods.

### Experimental design

A one-time basic application of phosphate fertilizer (6.0 g of calcium magnesium phosphate rock powder) was utilized. At the tillering stage and heading stage, 1.5 g of potassium chloride was applied to each barrel, and N fertilizer application was based on basal fertilizer:tiller fertilizer:panicle fertilizer = 5:3:2. The experiment was designed with three N application levels: low N (2 g according to pure N, 100 kg·hm^-2^), normal N (control, 4.5 g according to pure N, 225 kg·hm^-2^), and high N (7.5 g according to pure N, 375 kg·hm^-2^), and the source of N was urea. Drought stress and no drought were carried out for each N application level. The no-drought treatment: The rice was kept in a 2 cm water layer after transplanting. Drought stress: In the early stage of the no-drought treatment, from August 28 to September 3, the barrels were transported to the net room to dry naturally to approximately 10% of the soil moisture. A soil moisture meter was installed to monitor the soil moisture (measuring range 0 ~ 100%, WKT-M3, Jiangsu VicoMeter Instrumentation Co., Ltd., China), at this time the rice was at the heading stage. On September 3, the drought ended, and rehydration treatment (keeping the rice in a 2 cm water layer) was carried out. According to drought and different nitrogen levels, six treatments were set in the experiment: low N with no drought (LN), normal N with no drought (NN), high N with no drought (HN), low N with drought stress (LND), normal N with drought stress (NND), and high N with drought stress (HND). Each treatment was replicated three times, and 10 plastic barrels were used per replication. Overall, there were 30 parallel barrels with consistent growth conditions per treatment available for sampling. Thus, the 6 treatments had a total of 180 barrels.

### Determination of the SPAD value, dry matter, N content, and enzymatic activity

On the first day after the drought treatment, a SPAD-502 chlorophyll analyser (Zhejiang, China) was used to measure the SPAD value of the second bottom leaf of rice. Six rice plants with good and consistent growth were selected to measure the SPAD value of the base, middle and top of the leaf, and the average value was taken. At the heading stage, three rice plant samples were taken from each treatment and separated into the roots, stem sheaths, leaves and panicles. The dry matter samples were placed at 105°C for 30 min, dried to constant weight in an 80°C oven, and then weighed. The N content was determined by the Kjeldahl method (Sihite, 2022). The soluble protein content and nitrate reductase (NR), glutamine synthetase (GS) and soil urease activities were determined using kits from Suzhou Michy Biomedical Technology Co., Ltd. (Suzhou, China).

### Sample collection

Rice rhizosphere soil sampling: The entire rice root system and soil as a whole were excavated, the soil around the roots was removed, and only the soil on the roots was retained. A total of 10 g soil was obtained from each treatment, and three biological replicates were obtained from each treatment. Each sample was mixed.

Nonrhizosphere soil sampling: In the barrels from which the rice rhizosphere soil samples were taken, the soil was collected 10 cm from the plant to a depth of about 20 cm and put into a sampling tube. A total of 10 g soil was obtained from each treatment, and three biological replicates were obtained from each treatment. Each sample was mixed.

Rice root sampling: The whole rice plant was dug out with a shovel, the soil was washed with tap water, and root tips with a length of approximately 5 cm were taken. Three biological replicates were taken from each treatment.

All samples were first frozen in liquid N, transported to the laboratory and stored in a -80°C ultralow-temperature freezer until measurement.

### High-throughput sequencing

In this study, the V3-V4 region of the bacterial 16S rRNA (16S) gene was used as the target DNA sequence for PCR amplification. The V3-V4 region of 16S rDNA was amplified with the forward primer 343F (5’-TACGGRAGGCAGCAG-3’) and the reverse primer 798R (5’-AGGGTATCTAATCCT-3’). The PCR conditions were as follows: predenaturation at 95°C for 3 min; denaturation at 95°C for 30 s, annealing at 55°C for 30 s, and extension at 72°C for 45 s for 25 cycles, followed by extension at 72°C for 20 min. After amplification, the PCR amplification products were subjected to gel electrophoresis using 2% agarose to check the amplification effect. The PCR products of the samples were subjected to high-throughput sequencing on the Illumina MiSeq platform. The DNA extracting and 16S sequencing was performed according to the method of Zhu et al. (2022). 16S sequencing and analysis were conducted by OE Biotech Co., Ltd. (Shanghai, China). The 16S rRNA gene raw sequencing data from this study have been deposited in the Genome Sequence Archive in BIG Data Center (http://bigd.big.ac.cn/), Beijing Institute of Genomics (BIG) Chinese Academy of Sciences, under the accession number: CRA005709.

### Determination of rhizosphere soil metabolites

To determine the rhizosphere soil metabolites, 20 μL of an internal standard (L-2-chlorophenylalanine, 0.06 mg·mL^-1^; methanol configuration) and 1 mL methanol-water (V:V=1:1) were added to a 500 mg soil sample. Two small steel balls were added, placed at -20°C for 2 min to precool, and added to a grinder for grinding (60 Hz, 2 min), and the homogenized sample was transferred into a 15 mL centrifuge tube with 1 mL methanol-water (V:V=1:1). If the transfer tube wall remained, the above operation was repeated once. After centrifugation for 10 min (7700 rpm, 4°C), 2.5 mL of supernatant was placed in a 5 mL centrifuge tube, lyophilized, reconstituted with 400 μL methanol-water (V:V=1:4), vortexed for 60 s, sonicated for 30 s, and centrifuged for 10 min (12000 rpm, 4°C). A total of 150 μL of the supernatant was aspirated with a syringe, filtered with a 0.22 μm organic phase pinhole filter, transferred to an LC injection vial, and stored at -80°C.

The analytical instrument in this experiment was an LC-MS system composed of an ACQUITY UPLC I-Class plus ultrahigh-performance liquid phase tandem QE high-resolution mass spectrometer. Chromatographic conditions: column: ACQUITY UPLC HSS T3 (100 mm×2.1 mm, 1.8 um); column temperature: 45°C; mobile phase: mobile phase: A-water (containing 0.1% formic acid), B-acetonitrile (containing 0.1% Formic acid); flow rate: 0.35 mL·min^-1^; injection volume: 2 μL. Mass spectrometry conditions: ion source: electron spray ionization; sample mass spectrometry signals were collected in positive and negative ion scanning modes.

### 16S and metabolome analysis

16S analysis was performed according to the method of Zhu et al. (2022). Metabolome analysis was conducted by OE Biotech Co., Ltd. (Shanghai, China). The original LC-MS data were processed by Progenesis QI V2.3 software (Nonlinear Dynamics, Newcastle, UK) for baseline filtering, peak identification, integral, retention time correction, peak alignment, and normalization. The main parameters of 5 ppm precursor tolerance, 10 ppm product tolerance, and 5% product ion threshold were applied. Compound identification was based on the precise mass-to-charge ratio (M/z), secondary fragments, and isotopic distribution using the human metabolome database, lipid maps (V2.3), metlin, electron microscopy data bank, plant metabolome data bank, and self-built databases to perform qualitative analysis.

The extracted data were then further processed by removing any peaks with a missing value (ion intensity = 0) in more than 50% of groups, by replacing the zero value by half of the minimum value, and by screening according to the qualitative results of the compound. Compounds with resulting scores less than 36 (out of 60) were also deemed to be inaccurate and were removed. A data matrix was combined from the positive and negative ion data.

The matrix was imported into R to carry out principal component analysis (PCA) to observe the overall distribution among the samples and the stability of the whole analysis process. Orthogonal partial least-squares-discriminant analysis (OPLS-DA) and partial least-squares-discriminant analysis were utilized to distinguish the metabolites that differed between groups. To prevent overfitting, 7-fold cross-validation and 200-response permutation testing were used to evaluate the quality of the model.

Variable importance of projection (VIP) values obtained from the OPLS-DA model were used to rank the overall contribution of each variable to group discrimination. A two-tailed Student’s t test was further used to verify whether the differences in metabolites between groups were significant. Differential metabolites were selected with VIP values > 1.0 and *P* values < 0.05.

### Data analysis

Excel 2019 was used for data organization, Adobe Illustrator 2022 was used to integrate all figures. PCA and correlation heatmaps were drawn in the R language. SPAD, plant biomass, N content and accumulation, N-metabolism enzymes activity data were subjected to multivariate statistical Tukey’s test analysis with SPSS 18.0 software. In the multivariate analysis of microbial communities, ANOVA was used to compare the composition of microbial communities among treatments. The Chao1 index, Shannon index, Simpson index, 16S rRNA and environmental factor data were tested with Kruskal–Wallis statistical tests.

## Results

### Effects of different water and N conditions on the SPAD value of rice leaves

**Figure 1:**
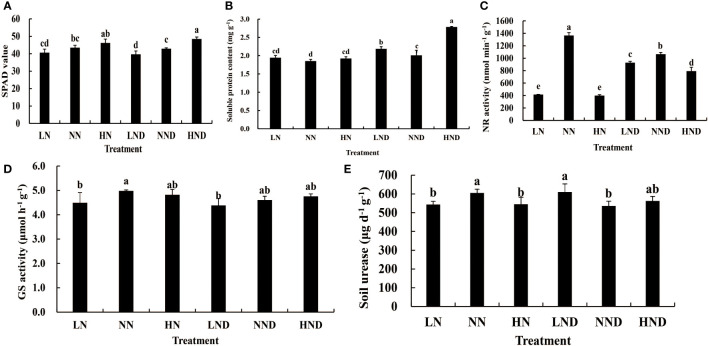
Effects of different water and N conditions on the rice leaf SPAD value and N-metabolism enzyme activities. **(A)** SPAD value; **(B)** soluble protein content; **(C)** NR activity; **(D)** GS activity; **(E)** soil urease activity. Lower-case letters represent the significance of the p-value at the 0.05 level.

Under no drought and drought stress, the SPAD value of rice leaves increased with increasing N application rate (**Figure 1A**). Under no drought stress, the SPAD value of HN was increased by 13.64% compared with that of LN, and there was a significant difference. Under drought stress, the SPAD value of NND was 8.18% higher than that of LND and reached a significant level. The SPAD value of HND was 12.97% higher than that of NND, and the difference was significant. The SPAD value of HND was 22.21% higher than that of LND, and the difference was significant.

### Effects of different water and N conditions on the dry matter mass of rice

There were differences in the dry matter mass of various organs of rice under different water and N conditions (**Table 1** and **Supplementary Figure 1**). Under no drought stress, the root dry matter mass of LN was 76.11% lower than that of NN. The root dry matter mass of HN was 27.99% lower than that of NN, and there was a significant difference. The stem sheath dry matter mass of NN was 61.52% higher than that of LN. The dry matter mass of leaves under NN increased by 109.88% compared with that under LN. The dry matter mass of leaves in HN increased by 43.92% compared with NN, and the difference was significant. Compared with LN, the panicle dry matter mass of NN increased by 122.78% and reached a significant level.

**Table 1:** Effects of different water and N conditions on the dry mass of various organs in rice.

Treatment	Root (g*·*plant-1)	Stem-sheath (g*·*plant^-1^)	Leaf (g*·*plant^-1^)	Panicle (g*·*plant^-1^)
LN	1.40c	9.85c	2.43d	3.38b
NN	5.86a	15.91b	5.10c	7.53a
HN	4.22b	20.51ab	7.34a	8.16a
LND	1.10c	6.39c	1.35d	2.54b
NND	3.35b	18.16ab	5.85bc	6.75a
HND	4.14b	21.97a	6.95ab	8.15a

Different lowercase letters indicate significant differences at P < 0.05. The same applies below.

Under drought stress, the dry matter mass of roots under LND decreased by 67.16% compared with that under NND, and the difference was significant. The dry matter mass of the stem sheaths in the LND treatment decreased by 64.81% compared with that in the NND treatment. The dry matter mass of leaves under LND decreased by 76.92% compared with that under NND, and the difference was significant. Under LND, the panicle dry matter mass decreased by 62.37% compared with that under NND.

### Effects of different water and N conditions on the N content of rice organs and soil

The N content of each organ of rice increased with increasing N application rate (**Table 2** and **Supplementary Figure 1**). In the no-drought treatment, the root N content of NN increased by 41.06% compared with that of LN. The root N content of HN increased by 54.18% compared with that of NN, and the difference reached a significant level. Compared with NN, the N content of the stem sheaths in HN increased by 44.18%, and there was a significant difference. The N content of leaves under NN increased by 15.56% compared with that under LN. The N content of leaves in HN increased by 20.99% compared with that in NN, and there was a significant difference. Compared with LN, the panicle N content under NN increased by 4.3%, and the difference reached a significant level.

**Table 2:** Effects of different water and N conditions on the N contents of rice organs and soil.

Treatment	Root (g*·*kg^-1^)	Stem-sheath (g*·*kg^-1^)	Leaf (g*·*kg^-1^)	Panicle (g*·*kg^-1^)	Soil (g*·*kg^-1^)	Rhizosphere soil (g*·*kg^-1^)
LN	2.63f	4.27c	16.90c	10.23cd	0.87a	0.83c
NN	3.71e	5.50b	19.53b	10.67bc	0.84b	0.85c
HN	5.72b	7.93a	23.63a	11.13ab	0.75d	0.90ab
LND	4.21d	4.49c	18.03c	9.81d	0.81c	0.86bc
NND	4.83c	5.32b	20.33b	11.13ab	0.75d	0.91a
HND	6.36a	8.12a	24.17a	11.83a	0.80c	0.83c

Lower-case letters represent the significance of the p-value at the 0.05 level.

Under drought stress, the N content of roots under NND increased by 14.73% compared with that under LND. The N content of roots under HND increased by 31.68% compared with that under NND, and the difference reached a significant level. The N content of the stem sheaths under NND increased by 18.49% compared with that under LND. Compared with NND, the N content of the stem sheaths in HND increased by 52.63%, and the difference reached a significant level. The N content of leaves under NND increased by 12.76% compared with that under LND. The N content of leaves under HND increased by 18.89% compared with that under NND, and there was a significant difference. Compared with that under LND, the panicle N content under NND increased by 13.46%, which was a significant difference.

Under different water and N conditions, the N contents of the rhizosphere soil and nonrhizosphere soil were also different (**Table 2**). Under the no-drought treatment, the N content of the nonrhizosphere soil decreased gradually with increasing N application rate. The N content of the nonrhizosphere soil under NN decreased by 3.45% compared with that under LN. The N content of the nonrhizosphere soil in HN was decreased by 10.71% compared with NN, and there was a significant difference. The N content of rhizosphere soil in HN was 5.88% higher than that in NN, a significant difference. Under drought stress, with the increase in the N application rate, the N content of the nonrhizosphere soil first decreased and then increased. The N content of nonrhizosphere soil in NND was 6.17% lower than that in LND. The N content in the nonrhizosphere soil under HND decreased by 6.67% compared with that under NND. The N content of the rhizosphere soil first increased and then decreased with increasing N application rate. The N content of rhizosphere soil in NND increased by 5.81% compared with that in LND. The N content of rhizosphere soil under HND decreased by 8.79% compared with that under NND, and the difference was significant.

Under the same N application rate, the N content of roots and leaves under drought stress was higher than that under the no-drought treatment.

### Effects of different water and N conditions on N accumulation in various organs of rice

Accumulated N is the product of dry matter mass and N content, and a certain differences in N accumulation also occur due to changes in the N content and dry matter mass in various organs of rice under different water and N conditions (**Table 3** and **Supplementary Figure 1**). In the no-drought treatment, the N accumulation of roots in LN decreased by 83.03% compared with that in NN, and the difference reached a significant level. The N accumulation of roots in HN increased by 10.09% compared with that in NN, although the difference was not significant. Compared with that under LN, the N accumulation in the stem sheaths under NN increased by 108.1%. The N accumulation of the stem sheaths in HN increased by 86.61% compared with that in NN, and there was a significant difference. The N accumulation in leaves under LN was 58.69% lower than that under NN. Compared with NN, the N accumulation of leaves in HN increased by 74.17%, and there was a significant difference. The N accumulation of the panicle under LN decreased by 56.98% compared with that under NN, and the difference reached a significant level.

**Table 3:** Effects of different water and N conditions on N accumulation in various organs of rice.

Treatment	Root (g*·*plant^-1^)	Stem-sheath (g*·*plant^-1^)	Leaf (g*·*plant^-1^)	Panicle (g*·*plant^-1^)
LN	0.37c	4.2c	4.11c	3.45b
NN	2.18ab	8.74b	9.95b	8.02a
HN	2.4a	16.31a	17.33a	9.07a
LND	0.47c	2.87c	2.41c	2.49b
NND	1.62b	9.61b	11.88b	7.5a
HND	2.62a	17.84a	16.79a	9.69a

Lower-case letters represent the significance of the p-value at the 0.05 level.

Under drought stress, the N accumulation of roots in LND decreased by 70.99% compared with that in NND. The root N accumulation under HND increased by 61.73% compared with that under NND, and the difference reached a significant level. Compared with NND, the N accumulation of the stem sheaths in LND decreased by 70.14%. The N accumulation in the stem sheaths under HND increased by 85.64% compared with that under NND, and there was a significant difference. Compared with NND, the HND plants accumulated 41.33% more leaves, and the difference reached significance. The N accumulation of the panicle in LND decreased by 66.8% compared with that in NND, and the difference was significant.

Under the same N application rate, the effects of drought stress on N accumulation in various organs of rice were different. Compared with the no-drought treatment, the N accumulation of roots in LND and HND increased by 27.03% and 9.17%, respectively.

### Effects of different water and N conditions on the activity of N-metabolism enzymes

Under different N application rates, the soluble protein content of roots under drought stress was higher than that under no drought stress to varying degrees (**Figure 1B**). The root soluble protein content of LND was 12.89% higher than that of LN. The root soluble protein content of NND was 8.65% higher than that of NN. The soluble protein content of HND was 45.31% higher than that of HN. Under the same N application rate, the root NR activity under drought stress was significantly different from that under no-drought treatment (**Figure 1C**). The root NR activity of HND was 99.12% higher than that of HN. The root NR activity of LND was 123.79% higher than that of LN. Under both drought stress and no-drought treatment, the NR activity of roots in NN was the highest, indicating that moderate N fertilizer can maintain higher NR activity. Compared with LN, the root GS activity of LND decreased by 2.45%. The root GS activity under NND was 7.63% lower than that under NN. The root GS activity of HND decreased by 1.45% compared with that of HN, although the difference was not significant (**Figure 1D**). The urease activity in the rhizosphere soil after drought stress was different from that under the no-drought treatment (**Figure 1E**). The rhizosphere soil urease activity of LND increased by 12.17% compared with that of LN, and there was a significant difference. The rhizosphere soil urease activity of HND increased by 3.35% compared with that of HN, although the difference was not significant. Compared with NN, the rhizosphere soil urease activity of NND decreased by 11.53%, and there was a significant difference.

### 16S analysis

**Figure 2:**
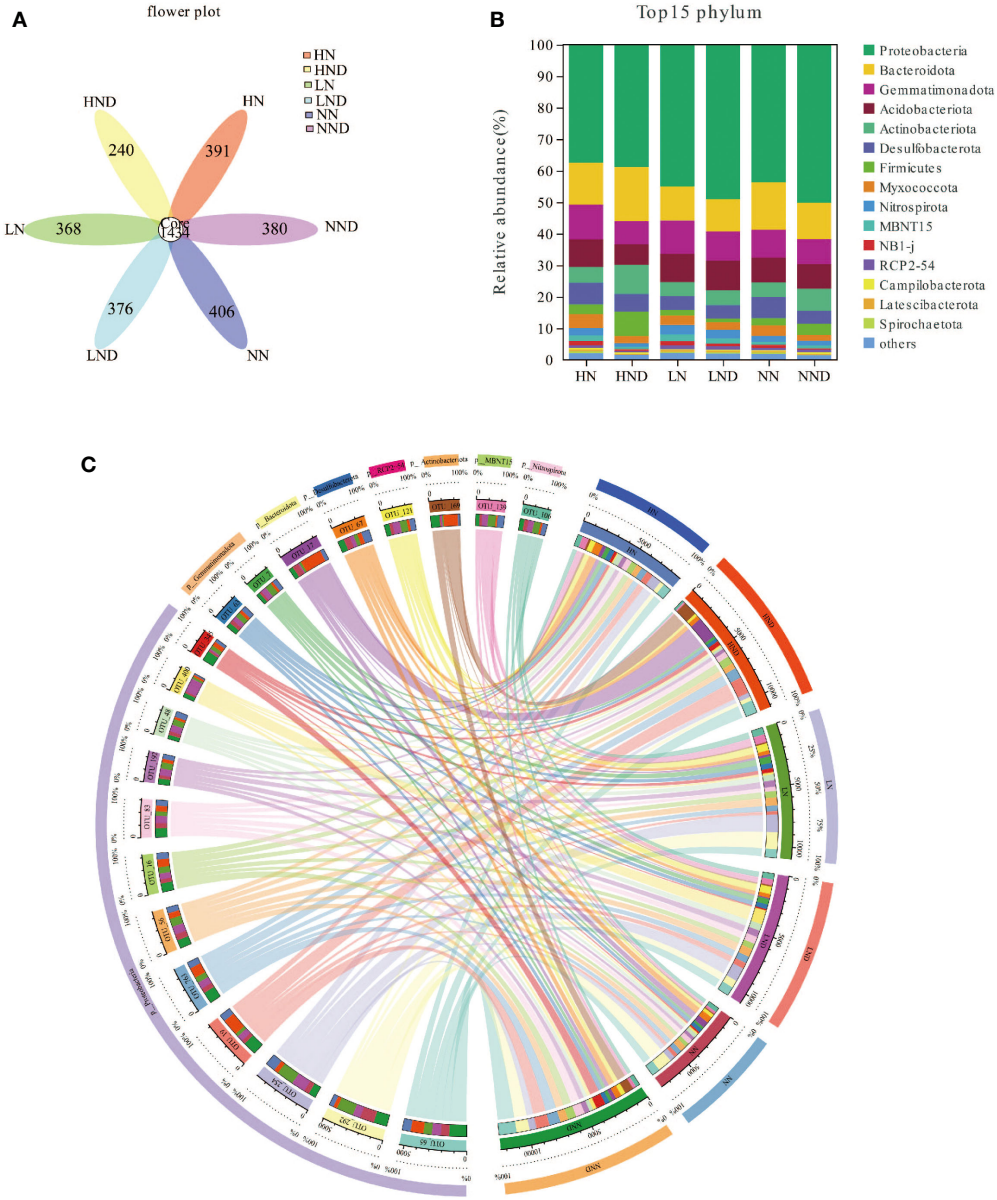
Root microbial community structure composition and diversity analysis. **(A)** Petal map of root microbial OTUs; **(B)** Bar plot of root microbial species composition changes at the phylum level; **(C)** Circos plot of root microbial species composition changes at the phylum level.

A petal diagram was drawn based on the overlap of sample OTUs for the analysis of shared and unique OTUs between different samples (**Figure 2A**). As seen from the figure, LN contained 1802 OTUs, including 368 unique OTUs; LND contained 1810 OTUs, including 376 unique OTUs. NN contained 1840 OTUs, including 406 unique OTUs; NND contained 1814 OTUs, including 380 unique OTUs. HN contained 1825 OTUs, including 391 unique OTUs. HND contained 1674 OTUs, including 240 unique OTUs.

The root microbial community composition was similar under different water and N conditions, but the proportion of various microorganisms was different. At the phylum level, Proteobacteria, Bacteroidota and Nitrospirota were the dominant groups, with HN accounting for 37.49%, 13.28%, and 2.40%, HND accounting for 38.88%, 17.17%, and 1.16%, LN accounting for 44.95%, 10.84%, and 3.03%, LND accounting for 49.03%, 10.24%, and 2.71%, NN accounting for 43.69%, 15.02%, and 1.92%, and NND accounting for 50.18%, 11.49%, and 1.52%, respectively (**Figures 2B, C**). Compared with the no-drought treatment, under the same N application rate, the proportion of Proteobacteria in LND was higher than that in LN. The proportion of Proteobacteria in NND was higher than that in NN. The proportion of Proteobacteria in HND was higher than that in HN, indicating that under the same N application rate, drought stress had a higher proportion. Drought stress was beneficial for increasing the abundance of Proteobacteria in the roots. Under the no-drought treatment, the proportion of Bacteroidota in NN was higher than that in LN and HN, indicating that the appropriate amount of N applied increased the abundance of Bacteroidota in the roots.

**Figure 3:**
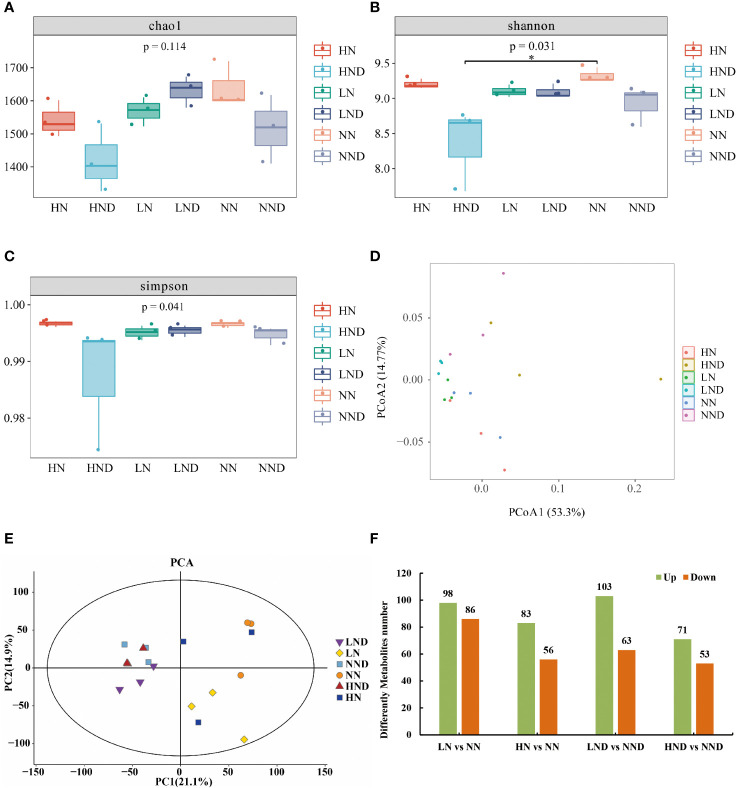
Root microbial diversity analysis, beta diversity analysis and up-and-downregulated DMs. **(A)** Chao1 index of the root microbial community; **(B)** Shannon index; **(C)** Simpson index; **(D)** PCoA of the root microbial community; **(E)** PCA of rhizosphere soil samples; **(F)** Up- and downregulated DMs. *p < 0.05.

We assessed alpha diversity between treatments using Chao1, Shannon and Simpson indices (**Figures 3A-C**). The results showed that there was no significant difference in the Chao1 index between LND and LN, NND and NN, and HND and HN under the same N application rate. The Shannon and Simpson indices of HND were significantly lower than those of HN, indicating that compared with the no-drought treatment, the high N treatment significantly reduced the microbial diversity of roots under drought stress.

We performed PCoA (principal coordinate analysis) on the samples (**Figure 3D**), and two principal components (PC1 and PC2) were extracted, i.e., 53.3% and 14.77%, respectively. The results showed that there were some differences among the samples. The closer the sample distance, the more similar the species composition. The farther the sample distance, the more different the species composition. Under the same N application rate, compared with the no-drought treatment, the distance between the samples in the HND was farther than that in HN, indicating that compared with the no-drought treatment, the high N treatment under drought stress had the most significant effect on the changes in the composition and structure of root microbial species.

### Metabolic profiling

Multivariate statistical analysis was performed on 18 rhizosphere soil samples by the PCA method, and the 18 samples were all within the 95% confidence interval. In the PCA score plot, two principal components (PC1 and PC2) were extracted, i.e., 21.1% and 14.9%, respectively (**Figure 3E**). The results showed that under different water and N conditions, there were certain differences between treatments. The drought treatments clustered at the left side but the N levels were not clearly separated. For the water treatments, the differences between nitrogen rates were much greater when the data points from HN and NN are mixed.

A total of 184 DMs (98 upregulated and 86 downregulated) were identified between LN and NN (**Supplementary Figure 2A** and **Figure 3F**). Among them were 29 fatty acyls, accounting for 15.76% (**Supplementary Table 1**). A total of 139 DMs (83 upregulated and 56 downregulated) were identified between HN and NN (**Supplementary Figure 2B** and **Figure 3F**). There were 29 fatty acyls, accounting for 10.79% (**Supplementary Table 2**). A total of 166 DMs (103 upregulated and 63 downregulated) were identified between LND and NND (**Supplementary Figure 2C** and **Figure 3F**). There were 15 fatty acyls, accounting for 9.04% (**Supplementary Table 3**). A total of 124 DMs (71 upregulated and 53 downregulated) were identified between HND and NND (**Supplementary Figure 2D** and **Figure 3F**). Among them were 14 fatty acyls, accounting for 11.29% (**Supplementary Table 4**).

### KEGG analysis

The Kyoto Encyclopedia of Genes and Genomes (KEGG) database is the main public database of metabolic pathways and can be used in studies of metabolite accumulation in a general network. In this study, we enriched the differential metabolites of each comparison group and classified them into different pathways (**Supplementary Table 5**). We found that arginine and proline metabolism were enriched in the LN and NN comparison groups. Amino sugar and nucleotide sugar metabolism were enriched in the LN and NN comparison groups and the LND and NND comparison groups. The phosphotransferase system (PTS) was enriched in the HN and NN comparison groups and the HND and NND comparison groups.

D-Alanine metabolism and phenylalanine, tyrosine and tryptophan biosynthesis were enriched in the HN and HND comparison groups, NN and NND comparison groups, and LN and LND comparison groups. This indicated that under the same N application rate, drought stress treatment had a certain degree of influence on D-alanine metabolism and phenylalanine, tyrosine and tryptophan biosynthesis in rhizosphere soil.

### Correlation analysis

**Figure 4:**
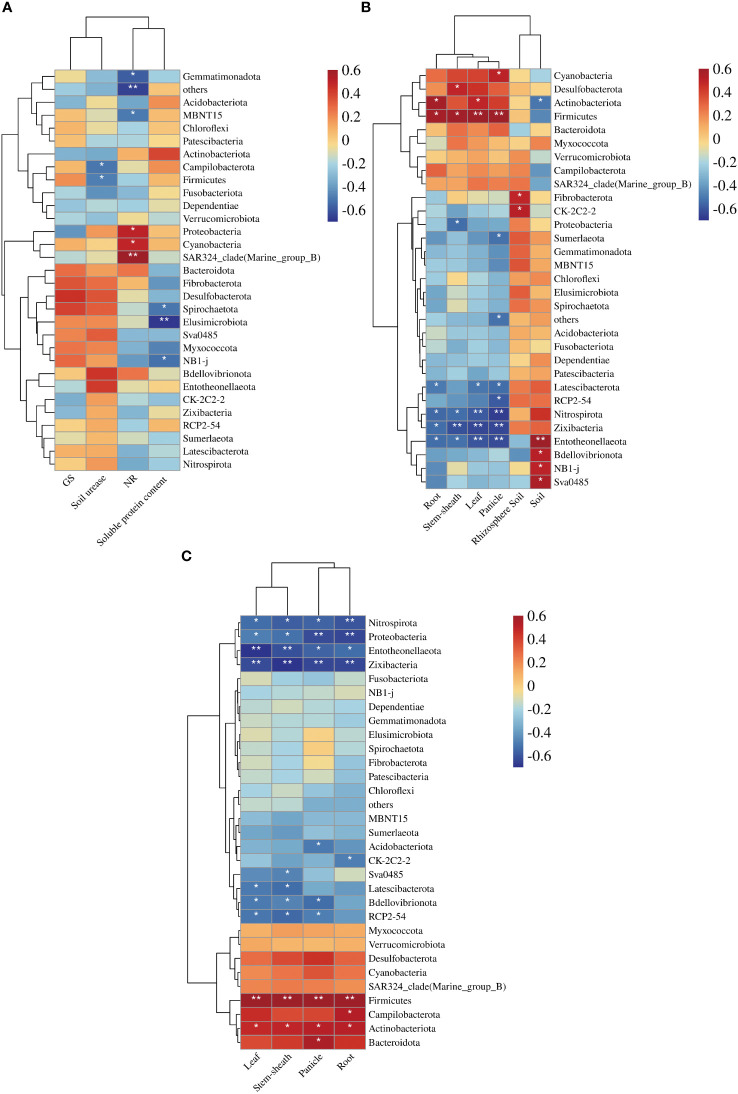
Correlation analysis between the environmental factors, N content, accumulation, dry matter mass and 16S rRNA. **(A)** Heat map of the correlation between 16S and N-metabolism enzyme activity; **(B)** Heat map of the correlation between 16S and N content; **(C)** Heat map of the correlation between 16S and N accumulation. Each grid represents the correlation between the two attributes, and different colors represent the size of the correlation coefficient between the attributes. *p < 0.05; **p < 0.01.

**Figure 5:**
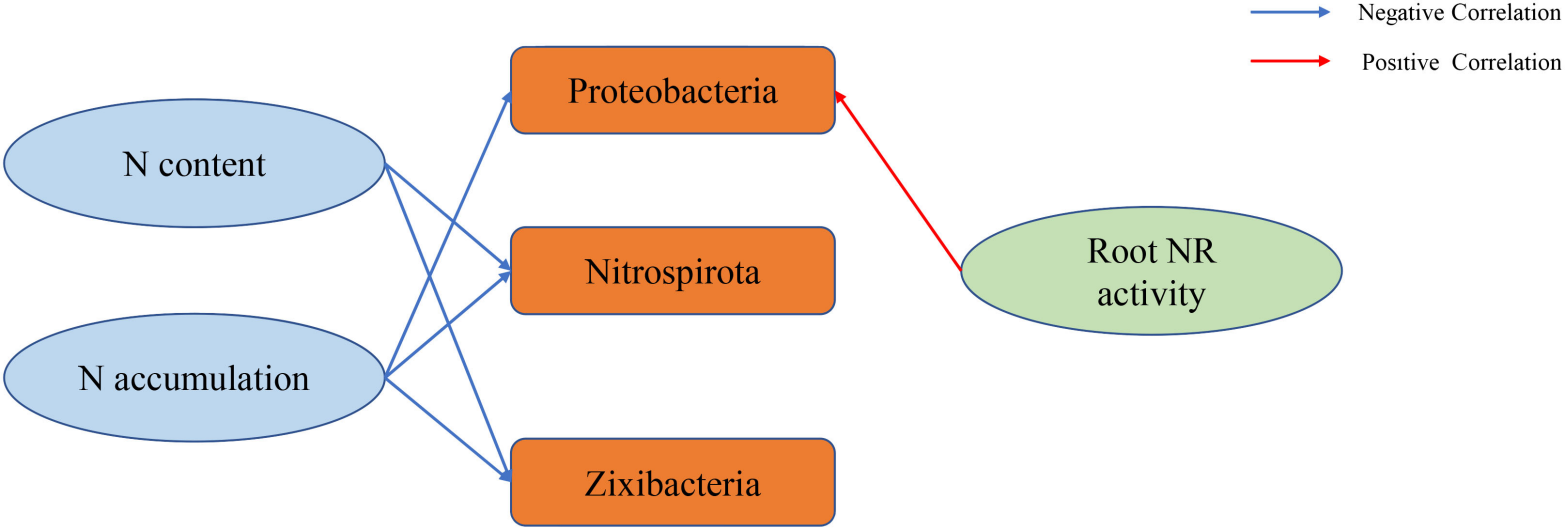
Flow chart visualizing the correlations between the N-metabolism enzyme activities, N content, accumulation and 16S.

We conducted a correlation analysis between environmental factors, N content, accumulation, dry matter mass and 16S rRNA. Root NR activity was significantly positively correlated with Proteobacteria (*P* < 0.05) (**Figure 4A**). The leaf N content, panicle N content, stem sheath N content, root N content, leaf N accumulation, panicle N accumulation, stem sheath N accumulation, and root N accumulation were significantly negatively correlated with Nitrospirota and Zixibacteria (**Figure 4B**). The N accumulation, panicle N accumulation, stem sheath N accumulation, and root N accumulation in leaves were significantly negatively correlated with Proteobacteria (**Figure 4C**). The correlation between N content, accumulation, N-metabolism enzyme activities and 16S rRNA is visualized more intuitively in **Figure 5**.

## Discussion

### Analysis of nitrogen uptake and 16S

Under the same N fertilizer level, the leaf SPAD value decreased with the decrease in soil water potential. Under the same soil water potential, the leaf SPAD value increased with the increase in N fertilizer level (Cheng et al., 2007). This study also showed that the SPAD value of rice leaves increased with the increase in N application under no drought and drought stress (**Figure 1A**). Dry matter is an important indicator for measuring crop yield, and moderate drought is beneficial to the increase in dry matter accumulation in rice (Yi et al., 2020) and the operation of dry matter in rice (Li et al., 2008). This study showed that the dry matter content of stem sheaths and leaves under NND increased to a certain extent compared with that under NN (**Table 1**). Water deficit can increase the air content in the plough layer soil, thereby increasing the soil redox potential, reducing the toxic substances, enhancing the root vigour, and enhancing the absorption of N by the roots (Liu, 2009). Mild water stress enhanced the ability of N redistribution and reuse in leaves and stem sheaths before heading, and it reduced N retention in stems and leaves, thereby improving N use efficiency and export efficiency (Wang et al., 2004). In this study, compared with the no-drought treatment, under the same N application rate, drought stress increased the N content of rice roots and leaves to a certain extent, and N accumulation in the stem sheaths of NND and HND increased to a certain extent (**Tables 2** and **3**). Nitrospirota is a key taxon that regulates N uptake by rice roots after abrupt drought-flood transitions (Zhu et al., 2022). One study found that the relative abundance of the phylum Zixibacteria was positively correlated with soil mineral N (Ihsan et al., 2022). The results of 16S analysis in this study showed that Nitrospirota was the dominant bacterium related to N absorption. The correlation analysis further showed that leaves N content, panicle N content, stem sheath N content, root N content, leaf N accumulation, panicle N accumulation, stem sheath N accumulation, and root N accumulation were significantly negatively correlated with Nitrospirota and Zixibacteria (**Figures 2B, C** and **4B, C**), which indicates that Nitrospirota and Zixibacteria had an important effect on N uptake in rice plants. Under drought stress, the content of soluble protein in the roots of rice seedlings increased rapidly and showed an upwards trend with increasing stress duration (Feng et al., 2020). The root NR activity of maize seedlings under drought stress was higher than that of the control (Si and Wu, 2001). A certain degree of drought stress enhanced root vigour, resulting in rapid root growth and increased secretions, which worked together with soil microorganisms to increase urease activity (Wang et al., 2020). In this study, under drought stress, root soluble protein, NR and soil urease activities increased to a certain extent (**Figures 1B, C, E**). At the same time, the results of 16S analysis showed that under the same N application rate, drought stress increased the root Proteobacteria abundance (**Figures 2B, C**). Proteobacteria have better drought resistance, especially with thicker cell walls, which are more resistant to water stress, explaining their obvious enrichment after drought stress (Wang et al., 2021). The correlation analysis also showed that root NR activity was significantly positively correlated with Proteobacteria (**Figure 4A**). Bacteroidota in the rhizosphere soil are easily enriched at the heading stage of rice (Chen et al., 2022). Appropriate N application can increase the abundance of Bacteroidota in rhizosphere soil. At the same time, Bacteroidota has also been identified as a highly N-sensitive bacterial group and a dominant phylum related to N absorption (Wang et al., 2022). Nitrogen addition usually inhibits microbial biomass, alleviating the effect of nitrogen limitation on some microorganisms, especially nitrifiers and denitrifiers that use inorganic nitrogen for energy or as an electron acceptor. A high level of N fertilizer can have a negative effect on the root microbial community (Liu et al., 2023). With the increase in nitrogen, some microorganisms with weak tolerance to high permeability potential may be killed, resulting in a decrease in microbial diversity (Xiong et al., 2021). This study also found that moderate N application was beneficial to the enhancement of Bacteroidota abundance in roots (**Figures 2B, C**). Therefore, moderate N application increased the bacterial diversity, and root bacterial abundance was increased under drought stress. This could provide a new idea for improving rice drought resistance.

### Analysis of metabolites and enrichment pathways

The LN and NN comparison group, HN and NN comparison group, LND and NND comparison group, and HND and NND comparison group had the most different metabolites in fatty acyls (**Supplementary Tables 1**-**4**), indicating that N application had a more significant effect on fatty acyls. Increasing the N supply can effectively increase the level of N-containing phospholipids, which in turn leads to the decomposition of diacylglycerols and triglycerides, thereby releasing more fatty acyl groups (Allwood et al., 2019). Amino acids, nitrogenous compounds and fatty acids were observed to be significantly different under different N supply levels. Most of them were increased by high N and decreased by low N (Xin et al., 2019). Arginine and proline metabolism and a one-carbon pool supplied by folate have been enriched after the addition of N fertilizer. Anthocyanin and flavonoid biosynthesis have also been enriched, meaning that N application was highly correlated with the synthesis of nutrients (Zhao et al., 2023). Low-N stress influenced the metabolism of arginine and proline (Hou et al., 2021). Rice N deficiency enrichment pathways are mainly associated with amino sugar and nucleotide sugar metabolism (Shen et al., 2019). In this study, arginine and proline metabolism were enriched in the LN and NN comparison groups. Amino sugar and nucleotide sugar metabolism were enriched in the LN and NN comparison groups and the LND and NND comparison groups (**Supplementary Table 5**). The phosphotransferase system (PTS) regulates N and carbon pools to maintain balance. For example, when N is limiting, the PTS system will work with the nitrogen stress response to enhance N uptake and metabolism (Hagberg et al., 2022). In this study, the HN and NN comparison groups and the HND and NND comparison groups were enriched with respect to the PTS (**Supplementary Table 5**). Amino acid sugars and nucleotide sugars are often used as glucose donors in the biosynthesis of various oligosaccharides and polysaccharides and are active forms of sugar synthesis or interconversion. Phosphoenolpyruvate: sugar PTS plays an important role in phosphorylating and transporting glucose in *Escherichia coli* (Zhou, 2010). This study found that under the same N application rate, drought treatment had a significant effect on D-alanine metabolism in rhizosphere soils and on phenylalanine, tyrosine and tryptophan biosynthesis (**Supplementary Table 5**). D-Alanine metabolism was highly enriched in the leaves of Camellia Tieguanyin plants under drought stress (Guo et al., 2017). Differentially expressed genes involved in phenylalanine, tyrosine and tryptophan biosynthesis were all upregulated specifically in a drought-tolerant Eruca line under drought stress (Huang et al., 2019). Energy metabolism pathways change strongly under abrupt drought–flood alteration stress (Xiong et al., 2019b). This study also showed that under drought stress and N application, the rhizosphere soil was mainly enriched in the energy metabolism pathway. Drought stress has a certain effect on the activity of N-metabolism enzymes in roots. The changes in the activities of N-metabolism enzymes further affected root microbial diversity and N absorption and utilization, thereby affecting the adaptability of rice to drought stress, which is of great significance for rice microbial drought resistance breeding.

## Conclusions

In this study, under the no-drought treatment and drought stress, the SPAD values of the rice leaves, dry matter mass of each organ, N content and N accumulation in each organ were positively correlated with the N application rate. Proteobacteria, Bacteroidota and Nitrospirota were the dominant flora related to root N uptake. There was a correlation between Proteobacteria and root NR activity and between Nitrospirota, Zixibacteria and the rice plant N content and N accumulation. Compared with the no-drought treatment, a certain degree of drought stress enhanced the N uptake and utilization capacity of the rice roots and stem sheaths. The activities of N-metabolism enzymes in roots also increased to varying degrees. Metabolites related to N uptake in rhizosphere soils were mainly fatty acyls. The results of KEGG analysis showed that energy metabolism pathways such as D-alanine metabolism, phenylalanine, tyrosine and tryptophan biosynthesis, arginine and proline metabolism, amino sugar and nucleotide sugar metabolism, and PTS were enriched in the rhizosphere soil under different water and N conditions. The comprehensive analysis of root N-metabolizing enzyme activities, microbial diversity and rhizosphere soil metabolites provides a new perspective on water and N regulation in rice breeding for drought resistance.

## Data availability statement

The original contributions presented in the study are publicly available. This data can be found here: NGDC, CRA005709.

## Author contributions

QX conceived and designed the experiment. GT, SC, HS, FL and JZ performed the data analysis. CS, HX and RW executed the experiment, analysed the data and interpreted the results. QX and CS wrote the manuscript. All authors contributed to the article and approved the submitted version.

## Supplementary material

Supplementary Figure 1 Phenotypic figures of 6 treatments at the heading stage. **(A)** LN; **(B)** LND; **(C)** NN; **(D)** NND; **(E)** HN; **(F)** HND.

Supplementary Figure 2 Rhizosphere soil differential metabolite volcano maps.

Supplementary Table 1 Identification information for DMs between the LN and NN treatments.

Supplementary Table 2 Identification information for DMs between the HN and NN treatments.

Supplementary Table 3 Identification information for DMs between the LND and NND treatments.

Supplementary Table 4 Identification information for DMs between the HND and NND treatments.

Supplementary Table 5 Enriched KEGG pathways associated with DMs.
