# Supplementary figures and images for: Integrated 16S and metabolomics revealed the mechanism of drought resistance and nitrogen uptake in rice at the heading stage under different nitrogen levels
Source: Front Plant Sci. 2023 Apr 4;14:1120584. doi: 10.3389/fpls.2023.1120584 (PMC10114610; doi:10.3389/fpls.2023.1120584)

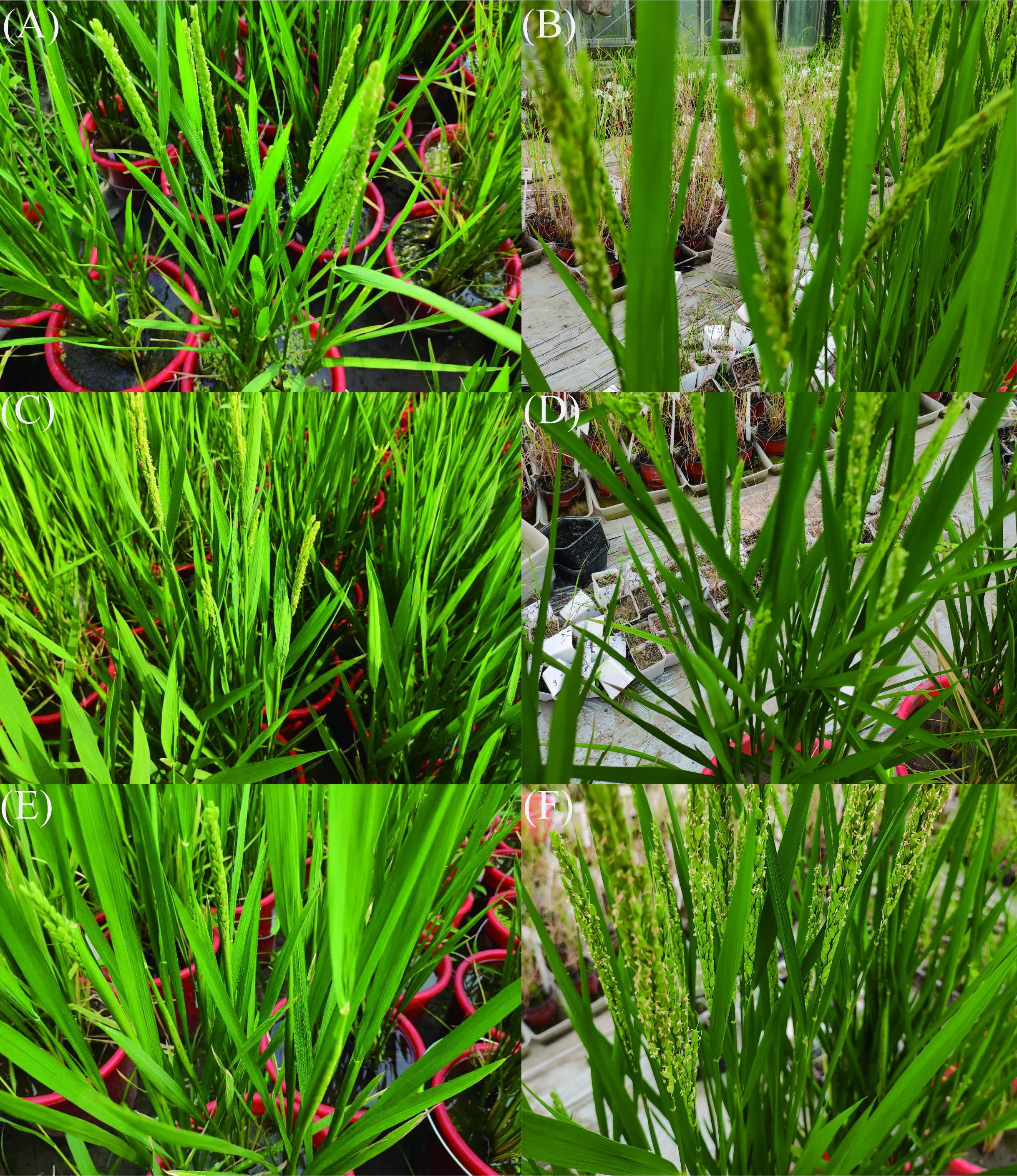

Supplement: Supplementary Figure 1 — Phenotypic figures of 6 treatments at the heading stage. (A) LN; (B) LND; (C) NN; (D) NND; (E) HN; (F) HND. [file Image_1.jpeg]

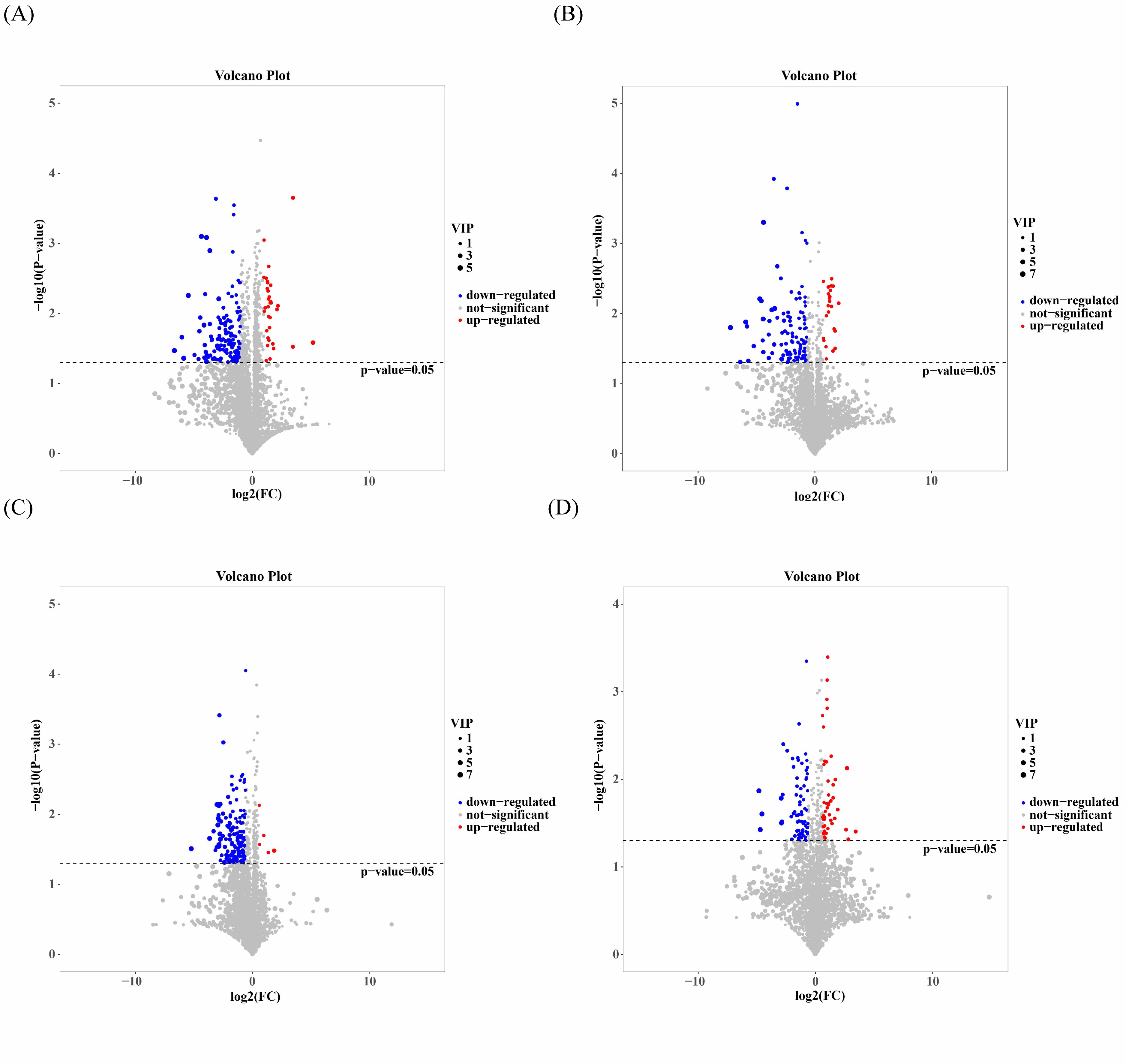

Supplement: Supplementary Figure 2 — Rhizosphere soil differential metabolite volcano maps. [file Image_2.jpeg]
