# Supplementary material for: Integrated 16S and metabolomics revealed the mechanism of drought resistance and nitrogen uptake in rice at the heading stage under different nitrogen levels
Source: Front Plant Sci. 2023 Apr 4;14:1120584. doi: 10.3389/fpls.2023.1120584 (PMC10114610; doi:10.3389/fpls.2023.1120584)
Supplement: Supplementary Table 5 — Enriched KEGG pathways associated with DMs. [file Table_5.docx]

**Supplementary Table 5.** Enriched KEGG pathways associated with DMs.

| Pathway Name | Pathway ID | Ratio_in_set | Ratio_in_background | p_value |
| --- | --- | --- | --- | --- |
| KEGG pathways between LN and NN | | | | |
| Glycolysis / Gluconeogenesis | ko00010 | 1/27 | 31/6095 | 0.1289 |
| Pentose phosphate pathway | ko00030 | 1/27 | 35/6095 | 0.1443 |
| Fatty acid biosynthesis | ko00061 | 1/27 | 56/6095 | 0.2210 |
| Arginine and proline metabolism | ko00330 | 1/27 | 78/6095 | 0.2943 |
| Amino sugar and nucleotide sugar metabolism | ko00520 | 1/27 | 108/6095 | 0.3835 |
| KEGG pathways between HN and NN | | | | |
| Arginine and proline metabolism | ko00330 | 2/19 | 78/6095 | 0.0240 |
| Amino sugar and nucleotide sugar metabolism | ko00520 | 2/19 | 108/6095 | 0.0437 |
| Valine, leucine and isoleucine biosynthesis | ko00290 | 1/19 | 23/6095 | 0.0694 |
| Protein digestion and absorption | ko04974 | 1/19 | 29/6095 | 0.0868 |
| Phosphotransferase system (PTS) | ko02060 | 1/19 | 50/6095 | 0.1451 |
| KEGG pathways between LND and NND | | | | |
| Valine, leucine and isoleucine biosynthesis | ko00290 | 1/16 | 23/6095 | 0.0588 |
| Glycolysis / Gluconeogenesis | ko00010 | 1/16 | 31/6095 | 0.0784 |
| Glycine, serine and threonine metabolism | ko00260 | 1/16 | 50/6095 | 0.1236 |
| Pentose and glucuronate interconversions | ko00040 | 1/16 | 56/6095 | 0.1375 |
| Amino sugar and nucleotide sugar metabolism | ko00520 | 1/16 | 108/6095 | 0.2490 |
| KEGG pathways between HND and NND | | | | |
| Lysine biosynthesis | ko00300 | 1/17 | 35/6095 | 0.0934 |
| Phosphotransferase system (PTS) | ko02060 | 1/17 | 50/6095 | 0.1308 |
| Biosynthesis of unsaturated fatty acids | ko01040 | 1/17 | 69/6095 | 0.1762 |
| Tryptophan metabolism | ko00380 | 1/17 | 83/6095 | 0.2082 |
| Protein digestion and absorption | ko04974 | 1/17 | 29/6095 | 0.0780 |
| KEGG pathways between HN and HND | | | | |
| D-Alanine metabolism | ko00473 | 1/42 | 8/6095 | 0.0407 |
| D-Glutamine and D-glutamate metabolism | ko00471 | 1/42 | 13/6095 | 0.0861 |
| Nitrogen metabolism | ko00910 | 1/42 | 19/6095 | 0.1233 |
| Phenylalanine, tyrosine and tryptophan biosynthesis | ko00400 | 1/42 | 34/6095 | 0.2100 |
| Arginine and proline metabolism | ko00330 | 1/42 | 78/6095 | 0.4189 |
| KEGG pathways between LN and LND | | | | |
| D-Alanine metabolism | ko00473 | 1/39 | 6/6095 | 0.0378 |
| Tyrosine metabolism | ko00350 | 2/39 | 78/6095 | 0.0884 |
| Phenylalanine, tyrosine and tryptophan biosynthesis | ko00400 | 1/39 | 34/6095 | 0.1966 |
| Pentose phosphate pathway | ko00030 | 1/39 | 35/6095 | 0.2017 |
| Lysine biosynthesis | ko00300 | 1/39 | 35/6095 | 0.2017 |
| KEGG pathways between NN and NND | | | | |
| D-Alanine metabolism | ko00473 | 1/50 | 6/6095 | 0.0482 |
| Phenylalanine, tyrosine and tryptophan biosynthesis | ko00400 | 1/50 | 34/6095 | 0.2448 |
| Lysine biosynthesis | ko00300 | 1/50 | 35/6095 | 0.2511 |
| Phenylalanine metabolism | ko00360 | 1/50 | 60/6095 | 0.3914 |
| Tryptophan metabolism | ko00380 | 1/50 | 83/6095 | 0.4976 |
